# Astroglial role in the pathophysiology of status *epilepticus*: an overview

**DOI:** 10.18632/oncotarget.25485

**Published:** 2018-06-01

**Authors:** Karina Vargas-Sánchez, Maria Mogilevskaya, John Rodríguez-Pérez, María G. Rubiano, José J. Javela, Rodrigo E. González-Reyes

**Affiliations:** ^1^ Biomedical Sciences Research Group, School of Medicine, Universidad Antonio Nariño, Bogotá, Colombia; ^2^ Universidad ECCI, Bogotá, Colombia; ^3^ Grupo de Clínica y Salud Mental, Programa de Psicología, Universidad Católica de Pereira, Pereira, Colombia; ^4^ Universidad del Rosario, Escuela de Medicina y Ciencias de la Salud, GI en Neurociencias-NeURos, Bogotá, Colombia

**Keywords:** astrocytes, status epilepticus, epilepsy, glia, neuroinflammation

## Abstract

Status epilepticus is a medical emergency with elevated morbidity and mortality rates, and represents a leading cause of epilepsy-related deaths. Though status epilepticus can occur at any age, it manifests more likely in children and elderly people. Despite the common prevalence of epileptic disorders, a complete explanation for the mechanisms leading to development of self-limited or long lasting seizures (as in status epilepticus) are still lacking. Apart from neurons, research evidence suggests the involvement of immune and glial cells in epileptogenesis. Among glial cells, astrocytes represent an ideal target for the study of the pathophysiology of status epilepticus, due to their key role in homeostatic balance of the central nervous system. During status epilepticus, astroglial cells are activated by the presence of cytokines, damage associated molecular patterns and reactive oxygen species. The persistent activation of astrocytes leads to a decrease in glutamate clearance with a corresponding accumulation in the synaptic extracellular space, increasing the chance of neuronal excitotoxicity. Moreover, major alterations in astrocytic gap junction coupling, inflammation and receptor expression, facilitate the generation of seizures. Astrocytes are also involved in dysregulation of inhibitory transmission in the central nervous system and directly participate in ionic homeostatic alterations during status epilepticus. In the present review, we focus on the functional and structural changes in astrocytic activity that participate in the development and maintenance of status epilepticus, with special attention on concurrent inflammatory alterations. We also include potential astrocytic treatment targets for status epilepticus.

## INTRODUCTION

Epilepsy encompasses a large group of complex neurological disorders characterized by the development of recurrent and usually unprovoked seizures [1]. According to recent estimates, epilepsy affects more than 50 million people worldwide [2]. This signifies that at any given time there are 4 to 14 people out of 1000 experiencing an active form of epilepsy. While epilepsy may manifest in any individual, its prevalence varies from country to country, ranging from 0.4 to 1% in developed countries to 1.03% (urban) and 1.54% (rural) in developing countries [3]. A substantial portion of epilepsy cases comes from low- and middle-income countries, where access to adequate treatment can be limited due to social, cultural, political and economic factors [4]. Although epilepsy can occur at any age independent of gender, there are several risk factors that can augment the chances of developing this disorder, including genetics [5], poor prenatal and perinatal care, poverty and malnutrition [6]. Epilepsy has a deteriorating influence on quality of life due to a number of factors such as depression, anxiety, body lesions related to seizures and adverse effects of treatment with anti-epileptic drugs [7, 8]. An epileptic disorder can manifest itself with diverse types of seizures classified as partial, generalized or unclassified nature [9]. While most seizures are self-limited, some can sustain for prolonged periods as happens in the particular case of status epilepticus (SE).

SE has been defined as a prolonged generalized tonic-clonic seizure persisting for more than 5 minutes (or 10 minutes for focal seizures with or without impairment of consciousness), or more than one seizure within a period of 5 minutes without recovery of consciousness in between [10, 11]. Occasionally, the first seizure of an individual develops into an SE, heralding the appearance of epilepsy. SE is a serious neurological condition and has to be treated as a medical emergency, as longer convulsive periods correlate with higher mortality. The acute mortality of SE varies from 7 to 39%, and approximately 20% of patients die within 30 days, with those surviving developing neurologic complications including epilepsy, encephalopathy and focal neurologic deficits [12, 13]. The neuronal damage produced by SE is frequently accompanied by cognitive changes such as attention deficit and hyperactivity disorder (ADHD), reduced information processing speed and perceptual-motor skills, and progressive cognitive decline [14–16]. SE is also a leading cause of epilepsy-related deaths accounting for up to 10% of lethal outcomes [10, 17–19].

Prevalence of SE among general population, according to different estimates, varies from 6.8 to 41 cases per 100.000 [18, 20–22]. This condition is most common in children (<10 years) and elderly people (>50 years), though it can still occur at any age [13, 23]. In fact, SE is considered as one of the most common neurological emergencies in childhood and has to be treated aggressively to prevent serious damage or death [24]. SE associated mortality rates strongly correlate with patient's age ranging from 3% in children to 38% in adults, and even up to 50% in patients older than 80 years [25]. Factors such as trauma, tumor, drug withdrawal, and infections, may contribute to the development of SE. However, an exact explanation of the pathophysiological mechanism that leads to a prolonged seizure is still needed. The fact that an SE seizure is not self-limiting and sustains during long periods of time can be explained by either failure in inhibitory mechanisms, continuous excitatory processes or both simultaneously [23, 26]. For example, alteration in the function of gamma-aminobutyric acid (GABA) or GABA receptors (inhibitory signaling) seems to be involved in the pathological mechanism of SE, as the conventional clinical treatment for SE consists of benzodiazepine treatment, which acts postsynaptically on GABA_A_ receptors, hyperpolarizing the neurons and improving the condition of the patient [27]. Even though some benzodiazepines show a successful outcome rate of up to 68% [26, 28] still some forms of SE are resistant to treatment with available medications [26].

An SE that does not respond to standard treatment regimens, such as an initial benzodiazepine followed by another anti-epileptic drug is defined as a refractory SE [17]. This condition occurs in about 30% of patients with SE and can result in significant morbidity [29]. Another classification of SE was introduced as super-refractory SE, defined as an SE that continues or recurs 24 hours or more after the onset of anesthesia, including those cases in which SE recurs on the reduction or withdrawal of anesthesia [30]. It is, therefore, of crucial importance to understand the basis of SE in order to seek novel and better treatment options for this dangerous condition.

The traditional neurocentric approach existing in clinical practice tends to neglect the functional dysregulation of other cell types that might facilitate generation of seizures. Recent research evidence suggests the involvement of glial and immune cells in epilepsy (reviewed in [31, 32]). Astrocytes, one of the most abundant and important cells in the central nervous system (CNS) [33], are known to participate in the immune response [34], and in the regulation of ion homeostasis [35, 36], as well as in the control of the concentration of various neurotransmitters including GABA [37] and glutamate (Glu) [38]. Due to these important functions, astrocytes are an interesting target for the comprehension of changes produced before, during and after a seizure, as well as the mechanistic elements that permit the development of an SE.

Astrocytes are highly reactive cells, constantly surveying the CNS environment and interacting with other cell types. Astrocytes become activated through pro-inflammatory cytokines released by microglial cells, damage-associated molecular patterns (DAMP) and reactive oxygen species (ROS) liberated from neurons, and in turn may initiate the release of cytokines [35, 39–41]. The aforementioned signals can induce reactive astrogliosis, a process referred to as changes in the expression of intermediate filament proteins, mainly through an increase in glial fibrillary acidic protein (GFAP) expression, as well as cellular hypertrophy [42, 43]. Astrogliosis is considered a hallmark of epilepsy [44] and inflammatory changes in the astrocytes can predispose to and facilitate the generation of seizures [35]. Inflammatory activity concomitant to SE results in morphological changes and protein expression alteration in astrocytes [39, 45]. Furthermore, activated astrocytes were shown to have decreased expression of excitatory amino acid transporters (EAAT), which transport Glu to the interior of the astrocyte, thereby exacerbating glutamate-induced neuronal cell death [46–48]. Moreover, astrocytic activation leads to an increase in intracellular calcium concentration, which results in intensification of Glu release as a gliotransmitter and further promoting excitotoxicity [49, 50]. Some brain regions seem to be more vulnerable to changes leading to excitotoxicity such as the hippocampus and this can be observed in patients with temporal lobe epilepsy (TLE) [51, 52]. Yet it is unclear which alterations in astrocytic activity lead to the development of self-limited seizures and which lead to the long-lasting propagating ones (as in the case of SE).

Thus, the aim of this article is to review state-of-the-art evidence on the role astrocytes may play in the development or maintenance of SE and the inflammatory changes that occur during and after SE in astrocytes.

### Reactive astrogliosis and SE

Astrocytes represent a heterogeneous population of glial cells concerned with the maintenance of homeostasis in the CNS. In order to accomplish this function, astrocytes are constantly monitoring the complex interactions between different cells (neurons, microglia, pericytes, and oligodendrocytes, among others) and exploring the environmental conditions of the neuropil, white matter and the blood-brain barrier (BBB) [33]. Thus, astrocytes exhibit different behaviors that allow them to protect and provide responses to different types of challenges present in the CNS, ranging from basic astrocyte activation, and reactive astrogliosis (defined above) to astrocyte proliferation [42]. A large scope of factors, including several of neuronal and glial origin, contribute to reactive astrogliosis (e.g. DAMP, ROS, ATP and Glu) as well as inflammatory signals (cytokines and chemokines), which may directly impact intracellular pathways such as the JAK-STAT pathway in astrocytes [53]. Reactive astrogliosis (also referred to as gliosis) is considered to be context (disease) dependent, multistage, region specific, diffuse or demarcating the injury site, and graded (from mild astrogliosis to glial scar) [54]. This response may also be adaptive (a defensive reaction aiming to restore homeostasis), or maladaptive (a persistent deleterious response). Once reactive astrogliosis is established, it can be further promoted through the release of cytokines by astrocytes acting as a feed-forward system [55]. Although it is still unknown which specific responses of astrogliosis are related to SE, some authors, as discussed below, have reported functional and morphological changes in astrocytes induced by either self-limiting seizures or SE activity.

Functional and morphological changes in astrocytes may vary depending on the etiology of seizure activity or SE and the brain region involved. For example, the International League Against Epilepsy (ILAE) classification presents 3 different types of hippocampal sclerosis and a gliosis-only (without neuronal loss) grouping [56]. The type 1 has extensive astrogliosis in all subfields (CA1, CA3 and CA4, sparring CA2) including the dentate gyrus, the type 2 has predominant gliosis in CA1, while the type 3 exhibits predominant gliosis in CA4. Types 2 and 3 have been reported to develop more frequently SE than other types, and also relate to an increased family history of epilepsy [57]. Although it is not clear if this augmented susceptibility for SE observed in types 2 and 3 is primarily due to astrogliosis or astrocytic dysfunction. Reactive astrogliosis is also known to be present in several types of medically refractory focal epilepsies including conditions induced by trauma, infections and ischemic injury, which could also lead to SE [41].

The functional and morphologic changes astrocytes undergo in the epileptic brain and during an SE may have a dual nature based on the underlying pathophysiological characteristics. On the one hand, reactive astrogliosis can occur as a compensatory mechanism following damage to the nervous system and lead to the reduction in excitability [58]. On the other hand, some components of reactive astrogliosis such as downregulation of aquaporin expression might have a direct epileptogenic effect [59]. Moreover, astrogliosis alone was shown to be sufficient to induce spontaneous recurrent seizures [60]. These ambiguous astrocytic responses may also be present during an SE.

Previous reports show that following an SE, reactive astrocytes become hypertrophic (increasing the expression of intermediate filament proteins), and develop longer and thicker processes [55, 61]. This initial astrocytic response could be part of glial compensatory and protective activity exhibited by these cells during CNS injuries. Though specific details of protective initial astrogliosis in SE have not been elucidated, evidence of similar beneficial responses in other conditions was previously demonstrated [62]. Astrocytes have a wide range of neuroprotective features including regulation of neuroinflammation, neurotransmitter response, oxidative stress and potassium extracellular levels [63]. Initial astrocytic response may aid to restore the homeostatic balance during a seizure by self-limiting the abnormal cellular activity. However, despite these neuroprotective efforts, the seizure activity is not halted in SE. Therefore, a critical control element is either altered or missing during the appearance or maintenance of SE. Although this precise element is currently not known, astrocytes should be considered as central agents in this process. Initial astrocytic reactivity in response to a seizure, may shift from protective to harmful, favoring the development of SE. As well, chronic astrogliosis could potentially alter the cellular microenvironment facilitating the initiation of SE. Widespread chronic astrogliosis, induced by conditional deletion of β1-integrin, caused the development of spontaneous seizures in a mouse model [60]. In addition, abnormal reactive astrogliosis may also compromise the function of the BBB, increasing its permeability and allowing the crossing of pro-convulsive agents to brain parenchyma. Other aspects related to astrogliosis and potentially involved in the appearance or maintenance of an SE are the loss of astrocytes and alterations in the intracellular domain organization of these cells. More research is needed in order to clarify the aforementioned mechanisms of the involvement of astrogliosis in SE.

Astrocytes may also behave different according to the time point of the SE. It has been observed that in early stages of SE there is no marked intensification in astrocytic proliferation, while at later stages, there is a rapid increase in the number of astrocytes [55]. Although astrocytes have the capacity to proliferate just after an SE, the reported small number of new cells suggest that in models of SE reactive astrocytes are comprised mainly of the resident astrocytes present before the insult [55, 64, 65]. It is still not clear what prompts this selective time course in the proliferative capacity of astrocytes regarding SE, or the functional and pathological implications of this response. A possible explanation may involve the phosphoprotein enriched in astrocytes of 15 kDa (PEA15), which is abundantly expressed in astrocytes. PEA15 is important for intracellular signaling and is involved in proliferation and prevention of apoptosis in astrocytes [66]. Phosphorylation of PEA15 at serine 104 (S104) site, activates cell proliferation through the ERK1/2 pathway [67], while PEA15 phosphorylation at serine 116 (S116) site prevents apoptosis by promoting the binding of Fas-associated death domain protein (FADD) to PEA15 [68, 69]. In a recent study with pilocarpine-induced SE, PEA15-S104 phosphorylation was unaltered in TUNEL positive rat astrocytes. Meanwhile, in the same publication, SE reduced astrocytic PEA15-S116 phosphorylation in the molecular layer of the dentate gyrus accompanied by massive loss of astroglial cells [70]. These findings may be useful to explain the reduced proliferative capacity of astrocytes at early stages of SE together with the increase in astrocytic apoptosis. Nonetheless, it is important to highlight that astrocytes represent a diverse population of cells, which may behave differently depending on the brain area or type of injury. Some populations of astrocytes may promote reactive astrogliosis while others may induce proliferation.

The expression of GFAP in astrocytes may also vary in a time-dependent manner in response to the presence of SE. In a rat model of lithium-pilocarpine-induced SE, a significant increase in GFAP in the hippocampus of the treated animals, compared with only lithium-treated controls, was observed 14 and 56 days after the injection with the drug, but not 1 day after [71]. The implications of increased expression in GFAP prompted by epileptogenic activity and SE are still not clear in humans, and it is unknown whether a sustained increased expression of GFAP alters the homeostatic regulation of astrocytes to facilitate the appearance or maintenance of SE. Although GFAP is a useful astrocytic marker for studying reactive astrogliosis, not all astrocytes express it [72, 73]. Exclusion of GFAP-negative astrocytes limits the analysis of astrocytic role in SE. Likewise, it is not clear what are the interactions between GFAP-positive (both non-reactive and reactive), and GFAP-negative astrocytes during seizures and SE.

The development of SE in rats alters the adequate function of mitochondria compromising the viability of astrocytes. This effect seems to be region-dependent, as SE induced astrocytic apoptosis together with decreased mitochondrial length in the molecular layer of the dentate gyrus, while mitochondrial elongation was observed in CA1 autophagic astrocytes [74]. In addition to the PEA15 activity mentioned above, another plausible explanation for SE-induced astrocytic death implicates the response to stress of mitochondrial cyclin-dependent kinase 5 (CD5K), since CDK5 phosphorylates the dynamin-related protein 1 (DRP1) promoting mitochondrial fission; furthermore, the use of CD5K inhibitors have shown to ameliorate astrogliosis and astroglial apoptosis during SE [75].

Alterations in the astrocytic expression of various proteins and receptors during astrogliosis such as voltage gated ion channels, neurotransmitter receptors and inflammatory cytokines are also prevalent in the epileptic brains of both human temporal lobe epilepsy (TLE) and animal models [55]. For example, studies of the hippocampus of patients with TLE revealed changes in the expression of genes known to be responsible for astrocytic structure and signaling including GFAP, ezrin, radixin and moesin (ERM) protein family, palladin, C-X-C chemokine receptor type 4 (CXCR-4) and the calcium-binding protein S100B [76]. It is still not clear if all these changes reported in TLE models are also present or induced by SE in astrocytes. Moreover, a number of functional and structural changes accompanying astrogliosis are not necessarily considered to be directly linked to epileptic activity and might happen independently.

Despite the gaps in knowledge, is clear that sustained seizure activity, as happens during SE, has a profound effect on astrocytes, prompting a reactive astrogliosis response with structural and functional changes that may even be sustained for long periods. The precise nature of this astrogliosis response, and whether it represents a beneficial compensatory mechanism or if it is part of the pathological processes responsible for the development of SE, remains to be elucidated. Other important aspect that need to be addressed in the future is how and to what extent this astrocytic response affects the whole brain cellular network and the brain barrier systems.

### Astrocytes and excitatory or inhibitory mechanisms leading to seizures and SE

Strong dysregulation of excitatory and inhibitory mechanisms has been considered for a long time to be a critical agent in the production of seizures and a hallmark of epileptic brains. More recently, it has been shown that astrocytes contribute to the aforementioned dysregulation [77]. Seizure activity is linked to elevated extracellular Glu levels and astrocytes are the major CNS cells responsible for Glu uptake from the synaptic cleft. In humans, an increase in Glu levels in the period between epileptic seizures was shown to be as high as 500% of the basal concentration [78, 79]. This suggests that the expression or the function of Glu transporters may be altered in the astrocytes of individuals with epileptic seizures and SE.

It has been reported that SE may induce TLE through neuronal hyperexcitability on Glu receptors, and this can be associated with alterations in the expression of Glu receptor subunit epsilon-2, also known as NMDA receptor subtype 2B (NMDAR2B or Nr2b), which induces an epileptic phenotype [80, 81]. Astrocytes have the potential to either increase or decrease epileptiform activity through the release of gliotransmitters including Glu, D-serine and ATP, which act directly on neurons. In addition, astrocytes regulate the expression of the neuronal N-methyl-D-aspartate receptor (NMDAR) subunits 2A and 2B and modulate cortical slow oscillations [82]. A study made in a mice model of pilocarpine-induced SE in which gliotransmitter release was genetically inhibited, showed that reduction in surface expression and function of neuronal NMDA receptors can delay seizure onset and attenuate subsequent progressive increase in seizure frequency, suggesting that astrocytes may be important in abating epileptogenesis [83].

Adenosine released by astrocytes is able to activate neuronal adenosine A1 receptors (A1R), which leads to phosphorylation of the NR2A and NR2B subunits of the NMDA receptor. This is mediated through activation of the Src family tyrosine kinases (SFKs) and decreases the rate of NMDA endocytosis [84]. However, if gliotransmition is inhibited, then both A1R activity and phosphorylation of NMDA receptor subunits are reduced, augmenting the rate of NMDA endocytosis, which in turn leads to a reduced surface expression of the NMDA receptor subunits. Therefore, astrocytic gliotransmission is involved in the surface expression of neuronal NMDA receptors.

The Glu ionotropic α-amino-3-hydroxy-5-methyl-4-isoxazolepropionic acid (AMPA) receptors are fundamental for the actions of Glu in the postsynaptic neurons. These receptors are responsible for the initial, sodium-dependent, rapid depolarization of postsynaptic neurons which leads to the dislodge of magnesium from the NMDA pore, allowing the activation of NMDA receptors, and the entry of calcium to the cell. Therefore, increased activity of AMPA receptors can be potentially dangerous to the neurons, augmenting the risk of excitotoxicity, epileptic activity and SE. Expression levels of AMPA receptors have also been shown to change in various animal models, and at least three different subunits of AMPA have been reported to change expression levels in the hippocampus after SE (GluR1, GluR2 and GluR3) [81, 85, 86]. For example, during SE, the expression of neuronal AMPARs containing GluA1 subunit are increased in hippocampal CA1 area [87]. Most of these changes have been studied in neurons and mostly focus in the expression (increased or decreased) of either AMPA or the functional consequences on postsynaptic neurons (for a more detailed review of changes in neuronal AMPARs during seizures and SE, see [81]). The relationship of astrocytes with AMPA in seizures and SE has not been as thoroughly investigated as with neurons. Astrocytes are able to express AMPA and astrocytes isolated from CA1 of patients with TLE have been shown to present a significant increase in flip splice variants of AMPARs, thus indicating a functional change in these receptors [88]. The direct implications on SE of these changes in astrocytic AMPARs are not clear. Also, is not known if astrocytes can directly induce an increase or decrease in neuronal AMPA expression and if this is somehow involved in the mechanisms responsible for halting epileptogenic activity (self-limited seizure), or in promoting long-lasting propagating signals, as in SE. Therefore, an important question that remains to be answered, especially in humans, is if abnormal astrocytic activation can lead to SE, through an increase in Glu release (as a gliotransmitter), promoting excessive postsynaptic activation of AMPA and subsequently NMDA receptors. Consequently, many aspects of the relationship between astrocytes and AMPA functionality during SE still need to be explored.

Under normal circumstances, the kainate receptor is not expressed in astrocytes, but it was shown in an SE-induced rat model, that astrocytes began to express the kainate subunits GluK1, GluK2/3, GluK4, and GluK5 in the CA1 hippocampal region, but not in the striatum, olfactory bulb or brainstem [89]. Even 8 weeks after the SE insult, significant levels of the kainate subunits were persistent in astrocytes. Astrocytic metabotropic Glu receptors (mGluR) may also be affected by the appearance of SE. In an experimental rat model of spontaneous seizures, the animals that developed SE showed an increase in the protein expression of mGluR2/3 and mGluR5 in the astrocytes of CA3 and the hilus, which persisted up to 3 months after SE [90]. These results suggest that, in astrocytes, both ionotropic and metabotropic Glu receptors could be altered before, during, or after an SE, although it is unknown which type of Glu receptor is related with which specific SE phase. The precise nature of these changes in Glu receptors in human SE is still not clear, as most studies have been conducted in animal models.

Astrocytic EAATs are primarily responsible for the removal of Glu from the extracellular space (doing so through Na^+^ and K^+^ gradients), hence preventing overactivation of Glu receptors and reducing the appearance of neuronal excitotoxicity [91–93]. Astrocytes express EAAT1 (GLAST1) and EAAT2 (GLT-1), and both were shown to have altered expression during epilepsy [94]. Traditionally, EAAT1 has been considered a predominantly “constitutive” transporter in astrocytes, while EAAT2 is strongly regulated by neuronal activity [95]. In addition, Glu transporter deficiency in mice has been shown to spontaneously develop seizure activity [46, 92, 96]. Knock-out mice for EAAT2 develop lethal seizures soon after birth [92] but, despite being expressed both in neurons and astrocytes, only the removal of astrocytic EAAT2 led to lower body weight, increased mortality and seizures in knock-out mice [97]. In a rat model of pilocarpine-induced SE, both EAAT1 and EAAT2, together with the NR1 subunit of the NMDA receptor, were found to be downregulated in the cortex during the latent period, while during the chronic period, EAAT2 remained downregulated in the hippocampus as opposed to NR1 which was reported to be augmented [98]. Interestingly, the same study reported no significant changes in EAAT1 mRNA expression levels in the hippocampus after SE induction. However, another study used an intrahippocampal kainic acid (IHKA) mouse model of TLE to evaluate GFAP, EAAT-2 and aquaporin-4 (AQP4) hippocampal expression at 1, 4, 7, and 30 days after SE induction [99]. The authors found an increase in the expression of EAAT-2 1 day post SE, a significant downregulation 4 to 7 days post SE, and a return to basal levels at 30 days post SE. Moreover, increased expression of EAATs was shown to be linked to diminished susceptibility to seizure development in the pilocarpine-induced animal model of epilepsy [100], thus suggesting the crucial importance of astrocytic Glu transporters in the control or generation of epileptic seizures.

In a kainic acid-induced SE model of TLE in rats, astrocytes were shown to clear Glu faster in brain slices from the treated animals compared with the controls, but without changes in the expression of EAAT1 or EAAT2, suggesting that other intrinsic properties of astrocytes could be involved such as modifications in connexin-astrocytic coupling [101]. In fact, evidence does not always suggest that EAATs are downregulated in epilepsy or SE. These other astrocytic adjustments may be present as compensatory mechanisms to prevent seizures and counteract hyperexcitability and excitotoxicity generated through enhanced Glu release. Furthermore, it has been shown that EAATs expression can vary from region to region and depend on the patient status [46, 102].

Once Glu is uptaken by the astrocyte, it can enter several pathways: a) it can be used by astrocytes as metabolic fuel by entering the tricarboxylic acid cycle (TCA) through Glu dehydrogenase (GDH) reaction, b) converted into glutamine (Gln) by glutamine synthetase (GS), or c) undergo *de novo* synthesis from glucose through intracellular metabolic pathways, followed by release in the form of gliotransmitter [93, 103, 104]. Neurons are dependent on astrocytes for Gln supply in order to maintain the Gln-Glu cycle [105]. Gln is synthesized in astrocytes in the reaction with Glu and ammonia, and then transported to GABAergic and glutamatergic neurons through system A type glutamine transporters (SAT) [106, 107]. Gln further serves for Glu and eventually for GABA synthesis in the neuronal cells [108]. In a recent Li-pilocarpine-induced SE study in rats, it was shown that hippocampal GS activity increases the first day after SE but decreases 14 days post-SE [71]. The same work showed an inverse relation for astrocytic GS content, decreasing at the first day post-SE, increasing at day 14 and reducing its levels again at day 56. These results suggest astrocytes are performing compensatory actions in order to remove the excess of Glu from the extracellular space, probably attempting to prevent the development of future spontaneous seizures. Hence, impairment of Gln synthesis or GS activity can considerably impact both excitatory and inhibitory neuronal transmission. Indeed, it was shown that in epileptic brains, the activity and concentration of GS is significantly decreased [109]. Likewise, chronic inhibition of GS activity in rats results in seizure generation [110]. Impaired function of GS can significantly impact intracellular concentration of Glu in astrocytes, ultimately slowing down the process of clearing Glu from the extracellular space [109]. Accumulation of ammonia due to GS function loss can also contribute to elevated extracellular concentration of Glu and induce neurotoxicity [111]. Furthermore, the activity of GDH was shown to be decreased in epileptic patients, which may also explain high extracellular concentrations of Glu in epileptic conditions [112].

Throughout epileptiform seizures, neurotransmitters can act on various astrocytic G protein-coupled receptors and induce increased calcium waves, leading to astroglial signaling [113, 114]. During SE, the Ca^2+^ oscillations in astrocytes may persist for 3 days, outlasting the period of epileptiform activity; this Ca^2+^ signaling may promote glutamate release from astrocytes, contributing to neuronal depolarization or Ca^2+^ elevations in neighboring neurons, increasing the risk of excitotoxicity and neuronal death [50, 115]. Studies performed on rodent pilocarpine models, showed that a few days after SE expression, the activity of T-type calcium channels isoforms were transcriptionally upregulated in the CA1 region of hippocampus but also in the thalamic nuclei, amplifying the hippocampal activity [116, 117]. In addition, astrocytic Ca^2+^ signaling induces the release of other agents including ATP, GABA and D-serine, which have been linked to SE [118, 119]. The relation between excessive Glu release, excitotoxicity and seizures has been reported amply, although it is not limited to epileptic disorders, as it has been observed in other diseases such as brain gliomas [120]. This emphasizes the crucial role astrocytes play in the control of Glu levels and the prevention of neuronal hyperexcitability.

GABA is the main inhibitory neurotransmitter in the CNS and alterations in GABA signaling have been proposed both in epilepsy and in SE. There is evidence that GABA released by interneurons can lead to activation of astrocytic GABA transporter GAT-3 [121]. This is a necessary process to regulate extracellular excessive GABA release. The activation of GAT-3 increases Na^+^ and eventually leads to an increase in Ca^2+^ concentration in astrocytes, prompting astroglia to release ATP/Adenosine which then acts on presynaptic adenosine receptors causing inhibition of neuronal glutamate release. The implications of astrocytic GAT-3 dependent heterosynaptic depression are still to be studied in SE. GABA uptake and GAT-3 expression may vary according to the brain region [122]. Evidence indicates a lower concentration of GABA in the hippocampus right before seizure onset, due to increase in GAT-3 expression [78, 123] (reviewed in [124]). Additionally, the activation of astrocytic GABA receptors induce depolarization of astrocytes by activation of voltage-gated Ca^2+^ channels which in turn increases intracellular astrocyte Ca^2+^ concentrations. However, endogenous GABA also can activate GABAb receptors and subsequently increase cytosolic Ca^2+^ which is released from intracellular stores [121]. Future studies should explore in depth the role astrocytes play in GABAergic regulation during an SE.

A recent study performed on a subset of astrocytes that express the Olig-2 marker, but not GFAP, showed that brain areas rich in these cells, strongly expressed GAT-3 in astrocytes and vesicular GABA transporter in neurons, suggesting Olig2-lineage astrocytes could be involved in inhibitory neuronal transmission [72]. Although this research was not conducted on an epilepsy or SE model, it implies a novel and interesting research approach comprising subtypes of GFAP-negative astrocytes, which could also help to clarify the role astroglia play in SE.

**Figure 1:**
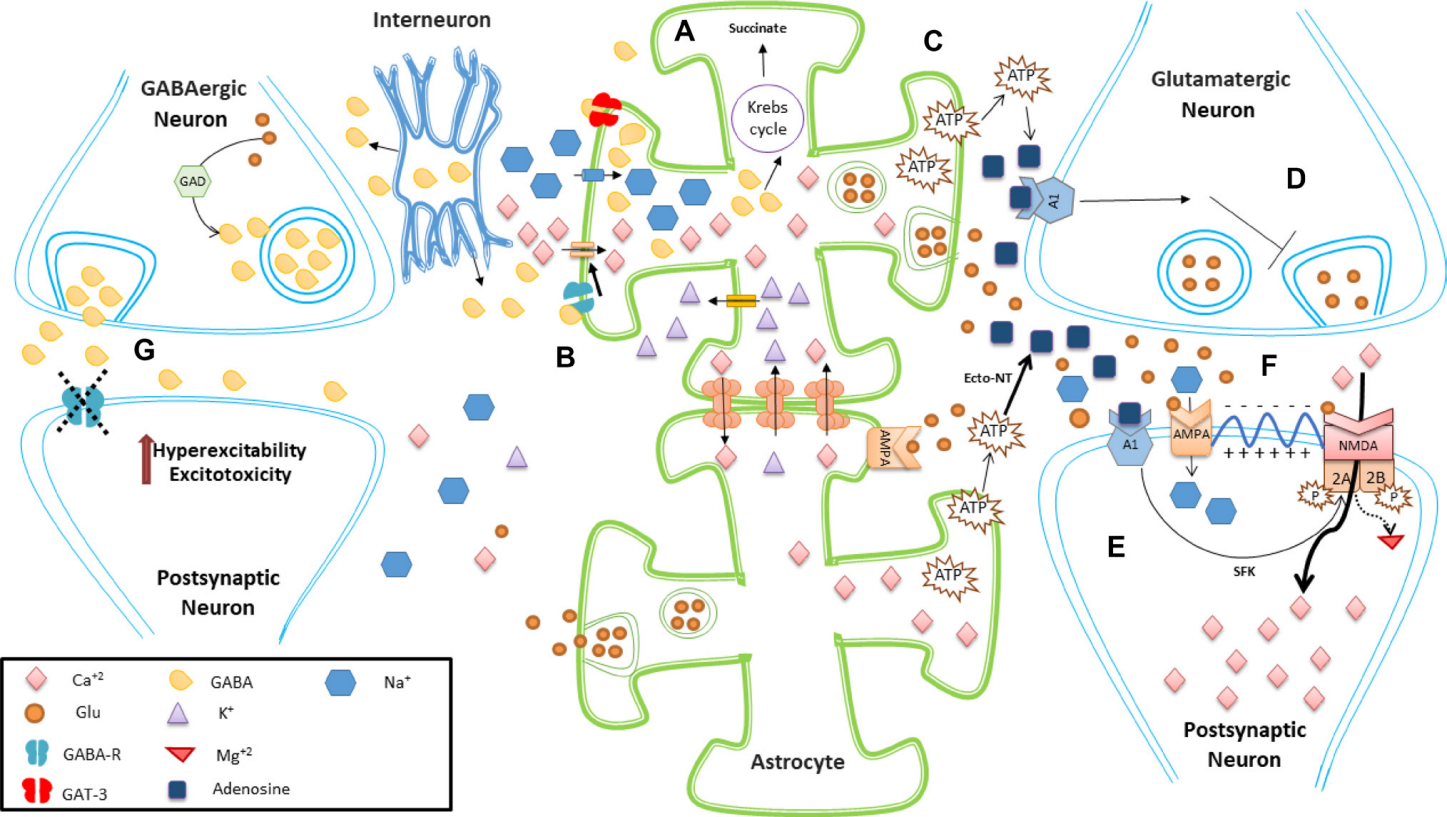
Schematic representation of excitatory and inhibitory effects of astrocytes (**A**) Astrocytes help to remove synaptic and perisynaptic GABA released from neurons, controlling GABA effects. In astrocytes, GABA can then be metabolized to succinate. (**B**) Activation of astrocytic GABA receptors induce depolarization of astrocytes by activation of voltage-gated Ca^2+^ channels, which in turn increases intracellular astrocyte Ca^2+^ concentrations. Furthermore, gap junction-coupled astrocytes can exchange Ca^2+^ with K^+^, helping to maintain both extracellular and intracellular homeostatic balance. (**C**) A rise in the intracellular calcium levels of astrocytes promotes the release of ATP, which in turn is converted into adenosine through the action of extracellular ecto-NT. (**D**) Adenosine acts on presynaptic neurons inhibiting the release of Glu. (**E**) Adenosine also acts on A1 receptors located in postsynaptic neurons, leading to phosphorylation of NR2A and NR2B subunits of NMDA receptor (mediated through SFK), reducing the endocytosis of these Glu receptors. Thus, astrocytic gliotransmission can help maintain the surface expression of postsynaptic NMDA receptors (**F**). Glu released from astrocytes can activate AMPA postsynaptic receptors, inducing neuronal depolarization and further removal of NMDA magnesium block. This allows the entry of calcium to the cell, which is important for many cell processes such as memory, but which also can induce excitotoxicity if not regulated properly. Astrocytes also express AMPA, but is not entirely clear how this may relate to SE. (**G**) Astrocytes convert Glu into Gln, which is transported into GABAergic neurons. Gln is converted into Glu and then into GABA thanks to the action of GAD. Under normal conditions, GABA can act on postsynaptic GABA receptors exerting inhibitory effects. Malfunction or blocking of these postsynaptic GABA receptors can lead to hyperexcitability, excitotoxicity or continuous epileptogenic activity. Abbreviations: α-amino-3-hydroxy-5-methyl-4-isoxazolepropionic acid receptor (AMPA); Ecto-nucleotidases (ecto-NT); gamma-aminobutyric acid (GABA); glutamate (Glu); glutamic acid decarboxylase (GAD); glutamine (Gln); N-Methyl-D-Aspartate (NMDA); Src family tyrosine kinases (SFK).

Despite the documented evidence describing the connection between astrocytes, Glu and GABA (Figure 1), there is still no definitive explanation as to how an excitatory or inhibitory mechanism leads to a self-limited seizure or a long-lasting one, as it happens in SE. Most studies on SE have centered on the effects of Glu dysregulation on neuronal post-synaptic receptors and astrocytes, but have not explored as thoroughly other neurotransmitters from an astrocytic point of view. More research both in the explanation of the basic physiological aspects of astrocytic function as in the mechanistic elements of SE are needed in order to clarify these points.

### Astrocytes and homeostatic alterations leading to seizures and SE

Under normal conditions, astroglial cells provide osmotic balance by regulating water and serving as a potassium buffer in the extracellular space. Maintenance of ionic homeostasis is crucial for the normal functioning of neuronal cells. During seizures, extracellular K^+^ augments and the prolonged neuronal activity is sustained [125], in part, by impaired astrocytic K^+^ clearance due to reduced inwardly rectifying potassium currents, and by an abnormal increase in astrocytic intracellular calcium concentration. Subsequently, after seizure termination, the extracellular potassium is reduced below baseline levels resulting in postictal depression [126]. Imbalance of ion gradients also result in deteriorated Glu reuptake followed by increased extracellular Glu concentration [127]. As stated in the previous section, an increase in the extracellular Glu concentration is critical for the development of excitotoxicity in SE.

In the epileptic brain, numerous factors lead to dysregulation in the K^+^ balance. First, various studies in the brain of epileptic patients have shown downregulation and aberrant functioning of Kir channels in astrocytes, also, Kir4.1 knockout animals develop spontaneous seizure activity [128–130]. Second, intensified neuronal firing occurring during epileptic seizures leads to an increase in the extracellular concentration of potassium [131]. Last, opening of the BBB during seizure activity allows extravasation of albumin which downregulates Kir 4.1 and Kir 3.1 expression in astrocytes [132]. These factors provide evidence, which links K^+^ dysregulation, astrocytes and the development of seizures and SE.

Astrocytic water influx is mediated through AQP4 channels expressed on astrocytic endfeet on perivascular space and colocalized with the astrocyte markers GFAP and S100B [133, 134]. Kir4.1 channels are co-expressed with AQP4 and therefore K^+^ influx is considered AQP4-dependent [133, 135]. Thus, AQP4 is fundamental for the regulation of rapid changes in the volume of astrocytes, and for the regulation of extracellular volume, necessary to modulate homeostatic adaptations involving K^+^ such as formation of edema and seizure susceptibility [133, 136]. In a pilocarpine-induced SE murine model, cytotoxic edema developed 3 hours after SE, followed by the development of vasogenic edema 2 days after SE [137]. The loss of AQP4 slows down K^+^ influx and predisposes to increased seizure severity, but also, impairment of K^+^ spatial buffering is known to associate with the development of epileptic conditions [61, 138]. Furthermore, decreased expression of AQP4 has been observed in the kainic acid model of epilepsy, with significant downregulation starting one day after SE and gradually increasing up to one month after SE [59, 99]. However, experiments in humans for chronic refractory TLE have shown that an increase in AQP4 expression is associated with astrogliosis and inflammation, in spite of reduction in Kir4.1 expression, which results in dysregulation of K^+^ homeostasis in the hippocampus [139]. These results are explained by dysfunction in the anchoring and localization of the AQP4/Kir4.1 complex to the astrocyte membrane due to decreased expression of dystrophin associated protein complex (DAPC) which is responsible for this anchorage. In addition, astrocyte swelling mediated by AQP4 can also increase Ca^2+^ concentration and signaling in astrocytes, which might lead to further triggering of gliotransmission, ATP release and initiation of an inflammatory response [140, 141]. Although the role AQP4 plays in the development or maintenance of SE is not clear, the homeostatic importance of astrocytes suggests a central role.

Ischemia and traumatic brain injury can lead to the development of cerebral edema, a condition frequently associated with the presence of pharmacoresistant seizures [142]. In this context, convulsive activity could be explained as a decrease in extracellular space osmolarity conducting to the appearance of edema in neurons, which generates slow inward currents (SICs) through non-synaptic activation of NMDA receptors and hyperexcitability [143]. These neuronal SICs occurred while astrocytes were in the process of swelling. Moreover, astrocytic swelling has been reported to increase calcium-dependent gliotransmitter release due to volume-sensitive organic anion channels activation in hippocampal CA1 neurons [143, 144]. Although the pathophysiological mechanisms of ischemia and brain traumatic injury are different from those of SE, both astrocytic activation and edema have been reported to be present in SE [145].

Connexin channels and gap junctions are the type of cellular contacts that allow the formation of the astrocytic syncytium. Gap junctions are important in the distribution of metabolites and signaling agents including Glu and potassium [146]. Several researchers have shown that upregulation of astrocytic connexins is linked to astrogliosis and epileptic conditions [101, 147–149]. In a mouse model of SE, the expression of connexins 40 and 43 were significantly increased in the dentate gyrus and in hippocampal CA1 and CA3 areas at 1 week and 2 months after the induction of SE [150]. It has been proposed that the increased gap junctional activity observed in astrocytes during SE may work as a compensatory mechanism buffering Glu and potassium levels [55]. However, some authors suggest that increased coupling of reactive astrocytes may be implicated in the synchronization of hippocampal hyperactivity leading to neuronal loss and epileptogenesis [150]. Furthermore, there is also evidence that deletion of astrocytic gap junctional proteins has pro-convulsive effects due to increased extracellular concentration of Glu and potassium [151]. In a mouse model of epilepsy, astrocytic gap junction uncoupling (appearing 4 hours after induction of SE), impaired potassium buffering and preceded apoptotic neuronal death and the generation of spontaneous seizures, as well, the authors showed that this astrocytic uncoupling could be induced in the presence of the pro-inflammatory cytokines IL-1β and TNF [152]. A reduction in the number of astrocytes, implicates a reduction in the functional capacity of astrocytic syncytium, and therefore may also be related to epileptogenesis and SE. This suggests that the metabolic and buffering activities of gap junction coupling between astrocytes are tightly balanced, and either excessive or reduced connectivity could induce the appearance or development of SE. Despite these results, there are still gaps in the knowledge about the role astrocytic gap junctions play in epilepsy and SE, and still is not clear if the changes involving astrocytic coupling (either compensatory or deleterious) observed in animal models are present in humans.

ATP can be released from astrocytes in a Ca^2+^ -dependent and -independent manner involving connexin channels [153]. ATP activates P2Y receptors inducing an excitatory signal mediated by the propagation of calcium waves [154]. In the extracellular space, ATP is metabolized to adenosine, which shows inhibitory (anticonvulsive) properties [155]. One of the potential underlying mechanisms of adenosine anticonvulsive effect is an activation of presynaptic A1 receptor by adenosine released by astrocytes [121]. Accruing data implies the connection of adenosine kinase (ADK) activity to astrogliosis occurring in the brain of patients with TLE. This evidence is supported by the observation of upregulated expression of ADK in the temporal lobe and hippocampus of TLE patients and, therefore, a lower concentration of adenosine [156]. ADK is notably increased in the cytoplasm of astrocytes in the 3–4 months following SE as shown on rats with progressive type of epilepsy [157]. Moreover, the link between astrocytic adenosine cycle and inflammatory processes was further highlighted by an experiment in cultured human astrocytes, which exhibited increased ADK expression in response to application of lipopolysaccharide (LPS) and IL-1β [156].

**Figure 2:**
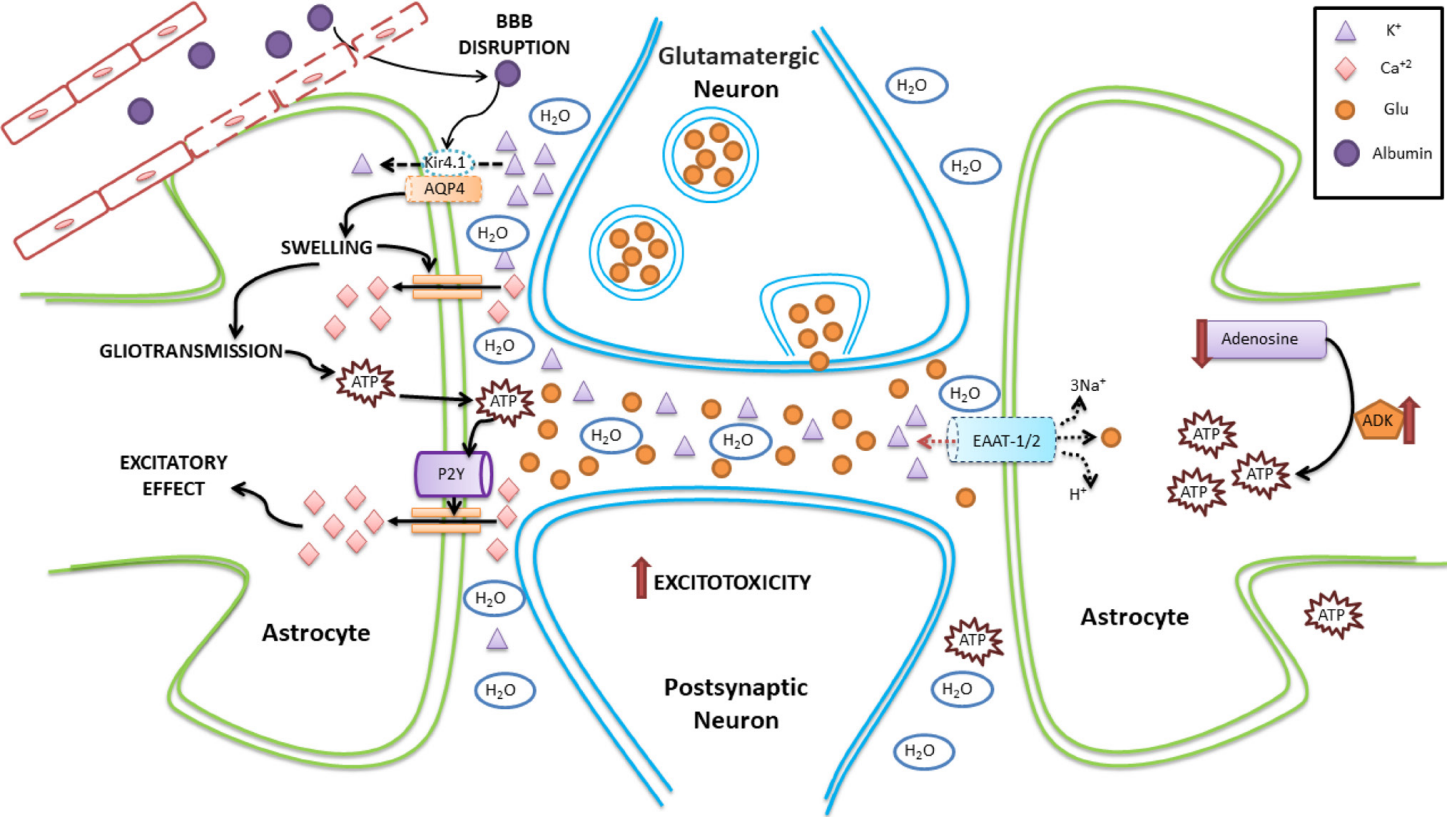
Schematic representation of homeostatic changes in SE involving astrocytes The disruption of the BBB allows the pass of albumin from the capillary to the brain parenchyma. Albumin affects the expression of AQP4, facilitating cell swelling and possibly edema. In addition, as Kir4.1 is coexpressed with AQP4, potassium is not transported efficiently into the astrocyte, accumulating in the extracellular space and altering neuronal excitability. Astrocyte swelling leads to an increase in intracellular calcium currents, which also alters gliotransmission. This favors the release of ATP from astrocytes, which activate P2Y receptors, further increasing the calcium astrocytic currents. In addition, there is a decrease in the synthesis of adenosine (which has anticonvulsant effects), an increase in the activity of adenosine kinase (which has proconvulsant effects), and an increase in the production of ATP. Dotted line represents either downregulation or inhibition.

Brain homeostatic balance depends on the activity of astrocytes. Many mechanisms involving astrocytes have been proposed to explain the development or maintenance of seizures, including potassium dysregulation, AQP4 rearrangement, brain edema, changes in gap-junction coupling and purinergic signaling, among others (Figure 2). Despite the reported evidence, there is no clarity about which temporal and spatial changes regarding the mentioned mechanisms could lead to SE development, and if any of those is present specifically during a SE or if it is shared with self-limited seizures. Other aspects that need to be addressed in the future are the effects of SE on the recently discovered glymphatic system and meningeal lymphatics.

### Astrocytes, inflammation and SE

Research evidence obtained over the last decade suggests that a variety of insults induce an astrocytic reaction in epilepsy, leading to initiation, regulation or amplification of immune responses [158, 159]. Dysregulated astrocytic immune responses may contribute to seizure development or play a role in events occurring after a seizure. Additionally, reactive astrocytes upregulate the expression of genes related to activation of innate immune/inflammatory responses in the areas of the brain where seizures originate and spread [41]. Therefore, astrocytic inflammatory activity could also be involved in SE.

Astrocytes, together with microglia, are the main cells involved in immune and inflammatory reactions in the CNS. Astrocytes are able to release both anti- and pro-inflammatory cytokines, and have been reported to produce elevated cytokine levels within 30 minutes from the onset of a seizure [160]. As well, astrocytes can react to the presence of cytokines. Studies based on an epilepsy mouse model reproducing chronic human mesial temporal lobe epilepsy with sclerotic hippocampus (MTLE-HS), demonstrated that impairment of astrocyte gap junction coupling starts early (within 4 h after SE), and was induced by the presence of IL-1β, TNF and LPS [152].

Astrocytes from epileptic tissues show overexpression of IL-1 type 1 receptor (IL-1R1) and IL-1β, suggesting the involvement of both autocrine and paracrine signaling [161]. During SE, IL-1β contributes to the activation of neurons in the hippocampus, and subsequently induce activation of astrocytes in limbic and extra-limbic areas (including thalamus), which start to express IL-1R1 transiently (from 6 to 18 hours) [162]. The aforementioned study failed to detect IL-1R1 immunostaining in microglia, despite the prominent microglial activation observed previously for this SE model [163]. A similar finding was reported on a mice model of pilocarpine-induced SE, where IL-1R1 was present in astrocytes but not in microglia [164]. These results may indicate astrocytes are more involved than microglia in the regulation of IL-1β during SE.

Elevated brain levels of IL-1β contribute to seizure activity. However, astrocytes also express IL-1RA (interleukin-1 receptor antagonist), which is an endogenous blocker of IL-1R1, and acts as an anticonvulsant, terminating or preventing the biological actions of IL-1β [165]. Despite this regulatory action, the upregulation of IL-1RA initiates with a several-hour delay, which suggests lessened efficiency of the brain in producing rapid protective responses towards sustained levels of IL-1β [166]. An alteration in the balance between the pro-inflammatory (IL-1RA and IL-1β) and anticonvulsant (IL-1RA) astrocytic actions may be present in SE.

Reactive astrogliosis contributes to the expression of IL-1R and its ligand IL-1β in astrocytes through activation of protein kinases and NF-κB signaling pathway [167]. In brain tissue from patients with medial temporal lobe epilepsy (MTLE) and hippocampal sclerosis in CA1, astrocytes (but not microglia) from injured areas overexpressed NF-κB-p65, suggesting that inflammatory processes are either chronically active or transiently re-induced by recurrent seizures [168]. Release of IL-1β potentiates cytokine gene expression, which in turn contributes to the onset and recurrence of spontaneous seizures lowering the neuronal excitability threshold [161]. Activation of IL-1R by IL-1β in astrocytes, decreases glutamate reuptake and simultaneously stimulates the release of glutamate from astroglial cells via TNFα induction, mediated through activation of TNFαR [169]. Alternatively, glutamate release might be augmented through IL-1β impact on NOS (nitric oxide synthase) activity; therefore, increased production of NO induces an inactivation of GS by nitrosylation [170]. Eventually, these events exacerbate neuronal excitability facilitating the appearance of epileptic seizures [166, 171, 172]. It is not clear if these same mechanisms involving astrocytes and IL-1β are also present in human SE.

IL-1β release is strongly related to TNFα expression from astrocytes, as both cytokines mutually initiate their transcription and release. TNFα affects excitatory transmission in hippocampus intensifying AMPA-dependent postsynaptic neuronal currents and lessening the strength of inhibitory transmission mediated by GABA. The former effect is due to indirect impact of TNFα on potentiation of neuronal calcium influx through involvement of AMPA receptors which lack the GluR2 subunit, while the latter inhibitory effect is attributed to the stimulation of GABA_A_ receptor endocytosis [166, 173, 174].

Persistent inflammation might have a deteriorating effect on cognitive function, which is commonly present in patients after the development of SE. Cytokine levels and the duration of their actions in the brain, determine the outcome of their effects on neuronal excitability. Animals whose astrocytes overexpress and release high levels of TNFα and IL-1β exhibit cognitive deficits and concurrent alterations in CNS excitability. In contrast, mice with low expression of TNFα exhibited resistance to the development of epileptic seizures [175, 176]. Astrocytic inflammatory responses are further amplificated by the expression of CCL2 (also known as monocyte chemotactic protein-1) and cyclooxygenase-2 (COX-2), which result in concurrent activation of IL-6, TNFα and transforming growth factor-beta (TGF-β) in seizures [41]. However, a pilocarpine model of TLE, shown that CCL4, but not CCL2, is overexpressed (together with its receptor CCR5) in astrocytes between 2 and 19 weeks after SE [177].

Astrocytes also express proteins of the complement system used to opsonize apoptotic cells (for example C1q, C3c, C3d), and provides them to phagocytic cells like activated microglia [178]. Aforementioned factors may trigger astrocytes to act as antigen presenting cells and promote brain inflammation by recruiting Th2 lymphocytes [41]. This effect could contribute to astrogliosis and the magnification of inflammatory responses observed in SE animal models and human brain tissue. Yet it remains to be elucidated whether the inflammatory response in astrocytes predispose to or directly follows the occurrence of epileptic seizures in SE.

Several studies have suggested that astrocytic DAMP and TLR overexpression is a basic feature of a lesion as observed in conditions such as traumatic brain injury (reviewed in [179]); however, repetitive seizure activity might intensify and aid to sustain astrocyte activation in a similar way. Studies in patients with mesial TLE (mTLE) shown that inflammatory gene expression can be involved in the modulation of seizure frequency [161, 180]. Increased expression of specific receptors of the TLR superfamily, specifically TLR4, were observed in sections of hippocampal tissue in the previous study. In astrocytes and neurons, this receptor is linked to high seizure frequency [181], supporting the role of TLR4 in epileptic seizures through amplification of the epileptogenic inflammatory signaling pathways. In contrast, evaluation of AMP-dependent transcription factor 3 (ATF-3), showed that reactive astrocytes lacked ATF-3 expression. The high expression of TLR4 and the suppressed expression of ATF-3 in patients with high seizure frequency implies that ATF-3 may work as a negative regulator of TLR4 signaling. Furthermore, the chemotactic chemokine IL-8 (CXCL8) is expressed in reactive astrocytes from human epileptic brain tissue, and also presents significant correlation with seizure frequency [182]. Both TLR4 and ATF-3 need to be further explored in different models of SE.

Post-seizure exposure of astrocytes to IL-1β in human epileptic tissue from patients with TLE, provoked the translocation of the high mobility group box 1 protein (HMGB1) from nucleus to the cytoplasm [183]. HMGB1 can be released as a DAMP signal, eliciting upregulation of TLR4 and proinflammatory mediators such as IL-1β and IL-1R1 in activated astrocytes and neurons [183–185]. Overexpression of IL-1β was shown in children with febrile seizures, however, those children that later developed febrile SE had as well increased levels of HMGB1 [186]. SE can initiate an escalation in the expression of major inflammatory mediators including IL-1R1 (for IL-1β), TLR4 and the Receptor for Advanced Glycation End product (RAGE) (for HMGB1), and prostaglandin E2 receptor 2 (EP2) (for PGE2), thus determining the prominent inflammatory signaling in the brain following SE [187–189]. The activation of RAGE by HMGB1 can induce the generation of seizures and initiate an innate immune response leading to inflammatory reaction in astrocytes [190].

The activation of TLR4 and IL-1R1 by HMGB1 in neurons and astrocytes, results in a rapid increase in NMDA receptor and calcium conductance via ceramide⁄Src-mediated phosphorylation of the NR2B subunit, leading to neuronal hyperexcitability [191]. This has a direct impact on neuronal excitability and in the generation and frequency of seizures. Indeed, the expression of an array of astrocytic genes involved in inflammatory responses was upregulated up to 1 week following SE [192], with the first mRNA expression being detected as early as 30 minutes after the SE [163]. At this same time point after the SE, the hippocampus and forebrain of adult mice and rats showed increased expression of IL-1β, TNF-α and IL-6, matching elevated levels of neuronal COX-2 [193]. The link between COX-2 in neurons and the initiation of inflammatory pathways was further highlighted by data from mice with genetic ablation of COX-2 gene in specific forebrain regions. In these transgenic animals, SE-induced production of cytokines and reactive gliosis was significantly diminished compared to a control group of wild-type mice [193, 194].

Changes in the BBB permeability have been reported in epilepsy and in SE [195]. BBB disruption and massive lymphocytic infiltration in patients with refractory SE, conduces to a pronounced focal inflammation, which in turn, induces intensive gliosis [196]. These findings suggest that opening of BBB is a factor delimiting the intensive gliosis and the appearance of SE. Vasogenic edema was shown to be induced by TNF-α-mediated NFκB activation in SE, doing so through the release and expression of endothelin-1 (ET-1) in endothelial cells from the BBB [197]. The binding of ET-1 to the endothelin B receptor (ETB), triggers signaling cascades to induce NOS activation and NO synthesis in endothelial cells. NO then disrupts the BBB and increases permeability through tight junction hydrolysis, mediated by metalloproteinases activation. ET-1-ETB signal contributes to increased formation of ROS such as NADPH oxidase, and subsequent astrocytic dysfunction [198]. Additionally, endothelial glutamate receptors can be activated during SE, which in turn leads to oxidative stress and BBB disruption [199].

Other markers related to inflammatory actions in astrocytes have been studied in SE. A recent study done in a mice lithium-pilocarpine-induced model of SE, documented a link between calcineurin overexpression, brain edema and reactive astrogliosis in SE [145]. In this work, calcineurin expression matched the time of brain edema occurrence and preceded astrocytic proliferation. As well, recent observations have shown that micromolar concentrations of the calcium-binding protein S100B, elicit neuronal and astrocytic death due to increased production of proinflammatory mediators, associated with S100B/RAGE [200]. The elevated concentrations of S100B causes neuronal death mainly through NO released from astrocytes, which result in up-regulation of COX-2 mediated by JNK/Cdc42/Rac1 and NFκB/Ras/Rac1 pathways [201].

**Figure 3:**
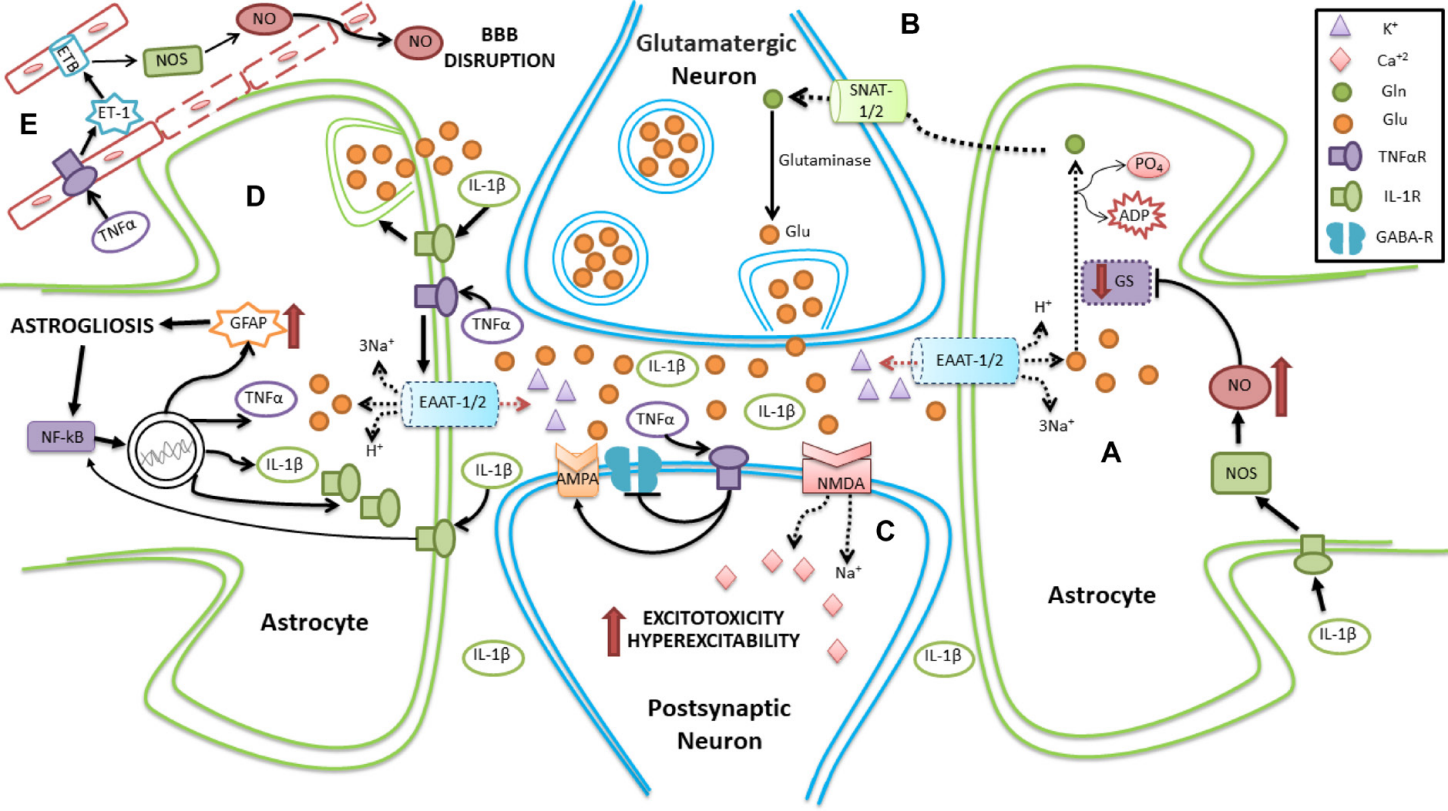
Schematic representation of neuroinflammation in SE involving astrocytes (**A**) Reduction in the reuptake of Glu is present due to decreased expression of EAAT1/2 transporters. Additionally, activation of IL-1R by IL-1β leads to inhibition of GS activity, thus diminishing the conversion of Glu to Gln. (**B**) Compromised Gln transport to the neuron leads to dysregulation in the formation and release of Glu from neurons. (**C**) An excess of circulating Glu acts on AMPA and NMDA receptors, producing hyperexcitability and excitotoxicity in the postsynaptic neuron. Moreover, TNF-αR activation by TNF-α, induces both an inhibition of GABA receptors and activation of AMPA, favoring hyperexcitability in postsynaptic neuron. (**D**) TNF-αR activation by TNF-α, reduces Glu reuptake. Furthermore, IL-1R activation by IL-1β promotes the release of Glu from astrocytes, and activates NFκB, which stimulates the production of pro-inflammatory agents. (**E**) Activation of TNF-αR on the capillary membrane induces an increase in ET-1, which activates its receptor and increases NO, disrupting the BBB. Dotted line represents either downregulation or inhibition.

Inflammatory changes are deeply involved in the mechanisms leading to the development of seizures and possibly in the initiation or maintenance of SE. Astrocytes are implicated in several aspects related to neuroinflammation including the production and release of cytokines and chemokines. Astrogliosis is a constant reaction observed in seizures and in SE, and is closely associated with inflammation. Furthermore, inflammatory agents directly affect metabolic and neuroenergetic functions of astrocytes (Figure 3).

### Astrocytes as potential treatment targets for SE

Astrocytes are now recognized as important players in the pathophysiology of epilepsy and SE. Astrocytes are the most important homeostatic regulators in the CNS, express numerous neurotransmitter receptors and transporters, release gliotransmitters, form the glymphatic system, are deeply involved in all the brain barrier systems, have immune and inflammatory functions, and frequently couple through gap-junctions with other astrocytes, generating an extensive functional syncytium. The current view on brain activity highlights the importance of the bi-directional interaction between astrocytes and neurons, as well as the interaction between astrocytes and other brain cells [202]. Astrocytes have been shown to modify the activity of neurons, in particular, regarding synaptic activity, through the release of gliotransmitters such as GABA (inhibitory signaling) or Glu (excitatory signaling), among others (reviewed in [203]), [204–207]. Therefore, therapies directed towards astrocytic targets might have a potential to treat SE or to complement existing treatment approaches. Hence, glial mechanisms involved in the interplay with neurons can serve as a therapeutic target for the treatment of epilepsy and SE.

In the epileptic brain, astrocytes and neurons exhibit hyperactive and overreactive features, including an increase in calcium oscillating frequencies. Some of these abnormal features have been successfully prevented with the administration of anticonvulsants such as valproate, gabapentin, and phenytoin [208, 209]. Actually, astrocytes have been shown to react to endozepines and to express receptors for benzodiazepines [210, 211]. Furthermore, the levels of benzodiazepine receptors in astrocytes augment under induced inflammatory brain damage [212]. Levetiracetam, one of the second-generation anticonvulsants, was shown to reduce inflammation, increase the expression of Cx43 and strengthen gap-junctions in astrocytes, restoring the membrane resting potential [213]. Currently, levetiracetam is being considered for the treatment of SE over more traditional medications like phenytoin [214]. Nonetheless, the effects of more recent medications, such as the third generation anticonvulsants lacosamide and eslicarbazepine acetate, have not been studied in astrocytes. Although the use of current anticonvulsants helps most patients, still a significant percentage (close to 30%) become refractory to treatment and susceptible to develop SE. The reasons behind this refractory behavior are usually not clear, and although several cells including neurons and microglia may be involved, the information presented in this review points astrocytes as relevant targets to be explored in this process. It is therefore necessary to understand the pharmacological interactions of current anticonvulsants with astrocytes and to develop new medications and therapeutic interventions that take into account astrocytes.

Another potential astrocytic target for antiepileptic therapy is adenosine together with its receptors (reviewed in [157]). Astrocytes are known to express different types of adenosine receptors such as A1R and A2AR [215]. Research directed towards an increase in adenosine was found to be effective in the suppression of seizures, including treatment-resistant forms such as SE. An agonist of the adenosine receptor A1R, demonstrated to inhibit spontaneous recurrent seizures in post-SE models [216]. An ADK inhibitor had a similar anticonvulsant effects in rodent and human cells [217]. Moreover, ADK expression levels in the brain might predict susceptibility to SE. In mice treated with an intra-amygdaloid injection of KA, those which overexpressed ADK presented aggravated symptoms of SE and higher mortality, while mice which expressed only 60% of normal levels of this enzyme, were completely resistant to KA injection convulsive activity [218]. A novel therapeutic approach may promote adenosine release and activity from astrocytes while reducing ADK effects.

Glu dysregulation is a major feature of epilepsy, as Glu can induce excitotoxicity, neuronal death and astrocytic swelling. Astrocytes are mainly responsible for Glu buffering, and under normal conditions, Glu is the major excitatory transmitter between neurons. Thus, it is important to look for novel mechanisms to reduce Glu toxicity during epilepsy and SE. Several approaches involving astrocytes can be taken, including improving the clearance efficiency of Glu from the synaptic cleft, allowing increased expression of Glu transporters, improving the efficiency of GS and therefore augmenting the release of Gln, and controlling the regulating of Glu metabolism in other aspects such as TCA and urea cycles.

Leptin and its receptors were shown to have dual effects in seizure generation [219, 220]. However, it was proposed that the controversial role of leptin signaling might be due to differential cell specific effects of this compound. Indeed, reactive astrocytes in epileptic mice upregulated leptin receptors and in *in vitro*, leptin was able to rescue astrocytes from Glu-induced cytotoxicity, possibly via faster conversion of Glu to Gln [221]. Therefore, leptin may also become a novel target for epilepsy and SE.

Reactive glial cells release various pro-inflammatory molecules including NOS, as mentioned previously [222]. NO continues to excite neurons and subsequently lowers seizure threshold after SE [223]. In a recent study, treatment of animals with a specific iNOS inhibitor (targeting exclusively glial NOS and not neuronal or endothelial) significantly decreased the number of incidents of recurrent seizures up to six months after SE [224]. Due to the important role astrocytes play in the brain neuroinflammation and the close relationship between inflammation and SE, the development of new therapeutics directed at attenuation or modulation of astrocytic inflammatory responses could prove helpful in the treatment or prevention of SE.

Seizures are energetically very expensive causing a significant increase in cellular energy consumption. During seizures, glucose metabolism is augmented, oxygen levels in the blood are decreased and glycolysis is increased [225]. Furthermore, a drop in the concentration of TCA intermediates may contribute to epileptogenesis. Therefore, one of the possible treatment options for patients suffering from epilepsy and at risk to develop SE, involves targeting of the metabolic pathways with ketogenic diet (KD), which has been in use for nearly 90 years. KD reduces glucose metabolism, increases fatty acids consumption and augments ketone bodies production. In astrocytes, these mechanisms reduce glycolysis and interfere with lactate formation, leading to less neuronal glutamate production and drop in seizures that help to prevent the development of SE [226–228]. Even though the KD response rate is close to 90% in metabolic regulation of seizure and SE, this diet is difficult to follow and, in consequence, other alternatives that can attenuate glucose metabolism are currently under evaluation. Some of these novel alternatives are directed at inhibiting lactate production and increasing seizure resistance [229, 230]. Thus, inhibition of lactate dehydrogenase (LDH) or the use of the glycolytic inhibitor 2-deoxy-D-glucose are mechanisms currently under evaluation to suppress seizures in animal models of TLE [226, 231].

Astrocytes can produce ketone bodies (KB), which have shown to offer a dual response regarding to Glu and GABA. On the one hand, KB downregulate excitatory synaptic transmission (suppressing Glu release), and on the other hand, upregulate inhibitory synaptic transmission (augmenting GABA levels) [232, 233]. Moreover, KB increase the ATP-sensitive potassium (K_ATP_) channel activity, which can lead to reduction of neuronal excitability during seizures [234]. Thus, KB reduce ATP synthesis and, therefore, the ATP available to inhibit ATP-sensitive potassium (K_ATP_) channels. This, in turn, activates and increases the potassium ion efflux helping to maintaining a negative resting membrane potential, and therefore reducing the chance of seizures and SE [226]. Another recently proposed mechanism to diminish glucose metabolism and activate K_ATP_ channels is associated with BAD protein deficiency or interference with its phosphorylation [226, 228, 235]. This targets both astrocytes and neurons and is aimed to to reverse seizure resistance. K_ATP_ channels are also expressed in astrocytes and its activation, especially the mitochondrial K_ATP_ channels, facilitate Glu uptake by Glu transporters in astrocytes, which exert neuronal protective roles [236]. Other possible additional benefits of KD include improve in mitochondrial function, and delay in both disease progression and the onset of severe seizures [237].

Oral treatment with triheptanoin, a tasteless triglyceride, had anticonvulsive effects in animals, increasing generalized seizure threshold and reducing the occurrence of absence seizures [238–240]. These results suggest astrocytes are involved in this process, as heptanoate and C5 ketones seem to be mainly metabolized by these cells. Use of triheptanoin in patients with various forms of epilepsy is now being studied in clinical trials [238]. Acetyl-L-carnitine (ALCAR) is another agent directed towards astrocytic metabolic pathways. ALCAR can be used by both neurons and astrocytes, however radioactive labeling suggested predominant astrocytic uptake [239]. Daily supplementation of ALCAR leads to upregulation in ATP and phosphocreatine levels [240, 241], and intraperitoneal administration in animals resulted in anticonvulsant effect [242]. Neuroenergetic regulation in astrocytes could be a powerful therapeutic tool in the prevention or control of SE.

Another novel therapeutic approach may involve the vascular endothelial growth factor (VEGF). This factor has been shown to be neuroprotective and protect hippocampal function in SE [243]. Astrocytes express the VEGF receptor VEGFR-1 (also known as flt-1) [244] and this factor have been shown to induce cell proliferation, gap-junction intercellular communication, and motility in astrocytes [245]. A recent study on adult male Sprague Dawley rats, showed that VEGF treatment significantly attenuated increases in hippocampal astrocyte size and complexity, but not in shape, caused by pilocarpine-induced SE [246]. Furthermore, the same investigation revealed that VEGF-treated animals demonstrated partial preservation of learning and memory in the Morris Water Maze (MWM), and that the changes observed in astrocyte size and complexity were significant predictors of this behavioral preservation.

Overall, as reviewed in this section, astrocytes present a broad range of targets for potential therapies against epilepsy and SE. In particular, regarding SE, these novel therapeutic efforts are extremely valuable in cases of refractory and super-refractory SE, which have high levels of morbidity and mortality and traditionally lack successful treatment options.

## CONCLUSIONS

It is difficult to overestimate the role of astrocytes in SE. Various studies have demonstrated pathophysiological alterations occurring in astrocytes linked to uncontrolled seizure generation and propagation. Following SE, astrocytes alter their morphology, proliferation and activation rate that subsequently induce changes in their responses against insults in the extracellular space, which can affect the brain parenchymal homeostasis. However, it remains unclear whether activation of astrocytes fulfills a compensatory function in the brain after a seizure, or if it represents a pathological mechanism linked to SE development.

Astrocytes also participate in dysregulation of inhibitory and excitatory transmission during SE. Simultaneous decrease in Glu uptake, impairment of Gln/GS activity and release of gliotransmitters by astroglial cells, have profound effect on both neuronal excitatory and inhibitory processes. Yet more studies are needed in this area to verify the causal relationship between varied processes occurring in astrocytes and morbid dysregulation of neuronal transmission in SE. Astrocytes might offer a protective effect in SE. Astrocytes can be prompted to release adenosine or GABA, both of which exert inhibitory actions over neuronal signaling. However, contribution of astrocytic GABA related responses still needs to be studied in depth in epilepsy and SE.

Glu concentration during SE is also altered by imbalance of ion gradients. During epileptiform activity, K^+^ is augmented in the extracellular space due to a diverse range of factors, including aberrant functioning of Kir and AQP4 channels in astrocytes. Even though the aforementioned alterations are linked to epileptogenesis, it is not clear whether they represent a unique feature of SE or are concurrent to all types of seizures.

Inflammatory responses in astrocytes are another prominent feature of SE. Astrocytes release and express a variety of proteins and molecular patterns implicated in immune responses including IL-1β, TNF-α, TLRs, complement proteins and DAMPs. However, data demonstrating whether astrocytic immune response is a characteristic of SE or plays a role in predisposing the brain to long-lasting propagating seizure is still missing.

Overall astrocytes represent a novel potential target for SE therapies. Nevertheless, more research is needed to pinpoint exact mechanisms and processes that malfunction exclusively during SE and directly affect seizure induction or propagation.
